# Editorial: The diversified role of mitochondria in cancer

**DOI:** 10.3389/fgene.2024.1494075

**Published:** 2024-09-27

**Authors:** Siyuan Song, Qingqing Xia, Yu’e Liu, Kuo Chen, Fengqian Chen, Jin Zhang, Yi Wang

**Affiliations:** ^1^ Department of Neuroscience, Baylor College of Medicine, Houston, TX, United States; ^2^ School of Medicine, University of Electronic Science and Technology of China, Chengdu, China; ^3^ Division of Hematology/Oncology, Boston Children’s Hospital and Dana-Farber Cancer Institute, Harvard Medical School, Boston, MA, United States; ^4^ First Affiliated Hospital of Zhengzhou University, Zhengzhou, China; ^5^ Cellphire Therapeutics Inc., Rockville, MD, United States; ^6^ Department of Pharmacology and Toxicology, University of Mississippi Medical Center, Jackson, MS, United States; ^7^ Center of Critical Care Medicine, Sichuan Provincial People’s Hospital, University of Electronic Science and Technology of China, Chengdu, Sichuan, China; ^8^ Clinical Immunology Translational Medicine Key Laboratory of Sichuan Province, Sichuan Provincial People’s Hospital, Chengdu, China

**Keywords:** mitochondrial dysfunction, cancer metabolism, immune evasion, programmed cell death, biomarkers, personalized therapy, tumor microenvironment, mitochondrial-targeted therapies

The role of mitochondria in cellular metabolism and disease progression has gained significant attention in recent years. Mitochondria, often referred to as the powerhouse of the cell, are not only essential for energy production but also regulate key processes like apoptosis, autophagy, oxidative stress, and immune responses. In cancer, these mitochondrial processes become dysregulated, contributing to tumorigenesis, metastasis, and resistance to therapy. Recent studies have shed light on how mitochondrial dysfunction influences cancer progression and opened the door for new diagnostic markers and therapeutic interventions.

One notable example is the role of choline dehydrogenase (CHDH), a mitochondrial enzyme that catalyzes the conversion of choline to betaine, a process crucial for the methionine cycle and cellular methylation. Alterations in CHDH expression have been associated with various cancers, including breast, pancreatic, and renal cancers, where single nucleotide polymorphisms (SNPs) in the CHDH gene can disrupt mitochondrial function and contribute to tumor progression. Beyond its role in cancer, CHDH is also linked to metabolic disorders such as male infertility and hyperhomocysteinemia, indicating its potential as a broad diagnostic marker. Furthermore, CHDH’s regulation of autophagy under cellular stress highlights its therapeutic relevance in conditions where mitochondrial health is compromised (Li et al.).

Another study underscores the impact of mitochondrial dysfunction in autoimmune diseases, specifically focusing on mitochondrial hub genes in dermatomyositis (DM). This autoimmune condition, characterized by muscle weakness and skin inflammation, has been shown to involve impaired mitochondrial function. Through bioinformatic analysis and *in vivo* validation, researchers identified three key mitochondrial-related genes (IFI27, CMPK2, LAP3) that are upregulated in DM. These genes are linked to oxidative phosphorylation and immune regulation, processes that are not only crucial in autoimmune diseases but also play a significant role in cancer biology. Mitochondrial dysfunction in DM might reflect similar mechanisms in cancer, where immune system dysregulation and energy metabolism lead to tissue damage and disease progression (Wang et al.). The overlap in mitochondrial pathways between cancer and autoimmune diseases suggests broader therapeutic targets for mitochondrial modulation.

Further highlighting the complex relationship between mitochondria and cancer, a review on non-coding RNAs (ncRNAs) and mitochondrial metabolism reprogramming in genitourinary cancers delves into how ncRNAs regulate mitochondrial function in cancers such as renal, bladder, and prostate cancers. The review discusses the role of ncRNAs, including miRNAs and lncRNAs, in influencing mitochondrial metabolism, oxidative phosphorylation, and glucose metabolism, pathways critical for cancer cell survival. Reprogramming mitochondrial metabolism allows cancer cells to thrive in nutrient-poor environments and evade cell death. Notably, specific ncRNAs such as PCA3 and UCA1 have been explored as biomarkers for prostate and bladder cancer, respectively, demonstrating their diagnostic and therapeutic potential. Targeting ncRNAs that influence mitochondrial function could lead to innovative treatments for genitourinary cancers (Thirunavukkarasu et al.).

In breast cancer, mitochondrial dysfunction is closely tied to programmed cell death (PCD)-related genes, which influence both disease progression and therapeutic outcomes. A recent study identified four key mitochondrial-related genes (CREB3L1, CAPG, SPINT1, GRK3) that are involved in regulating PCD and developed a prognostic model based on these genes. The model, validated in clinical datasets, demonstrated high accuracy in predicting survival outcomes for early-stage breast cancer patients. Interestingly, this study also explored the sensitivity of these patients to chemotherapy and found that those with higher mitochondrial dysfunction, as indicated by their gene signature, responded better to targeted therapies such as sorafenib and sunitinib. This suggests that mitochondrial dysfunction could be leveraged to tailor personalized treatment plans for breast cancer patients, improving overall survival (Wang and Jiang).

A comprehensive multi-center cohort study investigating mitochondrial transcriptome profiles in intrahepatic cholangiocarcinoma (iCCA) adds further evidence of the critical role of mitochondria in cancer. By integrating transcriptomic data from multiple cohorts, the researchers identified a five-gene mitochondrial signature (ANXA1, BCL2, GPT2, SNPH, and TUSC3) that predicts patient prognosis with high accuracy. This study also revealed that patients with higher mitochondrial dysfunction, as indicated by their mitochondrial score, exhibited lower levels of immune cell infiltration, including CD4^+^ T-cells and NK cells, suggesting that mitochondrial dysfunction contributes to immune evasion. The identification of two distinct molecular subtypes of iCCA, each associated with different survival outcomes, provides a framework for precision medicine, where treatment can be tailored based on mitochondrial function and immune characteristics (Chen et al.). Furthermore, combining mitochondrial-targeted therapies with immunotherapies could enhance treatment efficacy in iCCA.

These studies collectively demonstrate that mitochondrial dysfunction is a central player in cancer biology, influencing not only metabolic reprogramming but also immune responses and therapeutic sensitivity. By identifying mitochondrial biomarkers like CHDH and key ncRNAs, and linking them to cancer and metabolic disorders, researchers are opening new avenues for diagnostics and personalized treatments. The potential to target mitochondrial pathways in cancer treatment is particularly promising, offering a more nuanced approach to therapy that considers both the metabolic and immune landscapes of tumors.

However, while the findings are promising, they underscore the complexity of mitochondrial biology in cancer and other diseases. Future research must continue to unravel the intricate mechanisms through which mitochondrial dysfunction drives tumor progression and treatment resistance. Experimental validation in clinical settings and larger cohorts is crucial for translating these insights into actionable therapies. Furthermore, as the role of mitochondria in modulating immune responses becomes clearer, the development of combination therapies that target both mitochondrial function and the immune microenvironment holds significant potential.

In conclusion, mitochondria play a pivotal role in regulating cancer metabolism, immune evasion, and therapeutic responses. The identification of mitochondrial biomarkers and the development of prognostic signatures offer exciting opportunities for personalized treatment strategies. As our understanding of mitochondrial biology deepens, its integration into clinical practice promises to transform cancer treatment and improve patient outcomes across a range of diseases.

